# Expression of GCaMP6s in the dentate gyrus induces tonic–clonic seizures

**DOI:** 10.1038/s41598-024-58819-9

**Published:** 2024-04-06

**Authors:** Sasa Teng, Wanqi Wang, Jia Jun Joel Wen, Jingxuan Wang, Gergely F. Turi, Yueqing Peng

**Affiliations:** 1 Institute for Genomic Medicine, Vagelos College of Physicians and Surgeons, Columbia University, New York, NY 10032 USA; 2 Department of Pathology and Cell Biology, Vagelos College of Physicians and Surgeons, Columbia University, New York, NY 10032 USA; 3 Department of Genetics and Development, Vagelos College of Physicians and Surgeons, Columbia University, New York, NY 10032 USA; 4 Columbia College, Columbia University, New York, NY 10027 USA; 5 Division of Systems Neuroscience, New York State Psychiatric Institute, New York, NY 10032 USA; 6 Department of Psychiatry, Vagelos College of Physicians and Surgeons, Columbia University, New York, NY 10032 USA

**Keywords:** Seizures, Dentate gyrus, GCaMP6s, Ca^2+^, EEG, Epilepsy, Hippocampus

## Abstract

GCaMP is a genetically encoded calcium indicator (GECI) widely used in neuroscience research. It measures intracellular Ca^2+^ level by fluorescence changes as it directly binds to Ca^2+^. In this process, the effect of this calcium buffer on the intracellular calcium signaling and cell physiology is often not taken into consideration. However, growing evidence from calcium imaging studies shows GCaMP expression under certain conditions can generate aberrant activity, such as seizures. In this study, we examined the effect of GCaMP6 expression in the dentate gyrus (DG) on epileptogenesis. We found that viral expression of GCaMP6s but not GCaMP6f in the DG induces tonic–clonic seizures several weeks after viral injection. Cell-type specific expression of GCaMP6s revealed the granule cells (GCs) as the key player in GCaMP6s-induced epilepsy. Finally, by using slice electrophysiology, we demonstrated that GCaMP6s expression increases neuronal excitability in the GCs. Together, this study highlights the ability of GCaMP6s in DG-associated epileptogenesis.

## Introduction

The Dentate Gyrus (DG) is a hippocampal subarea which is highly conserved across mammals^1^. It plays an important role in learning, memory^2^, and is one of the few places in the brain where new neurons are generated throughout the lifetime^3,4^. The DG is made up of three layers: the molecular layer, granule cell layer, and the polymorphic layer. The molecular layer is composed of the dendrites of dentate granule cells (GCs) and axonal collaterals originating from the entorhinal cortex. The granule cell layer contains the tightly packed cell bodies of glutamatergic GCs and other various interneuron subtypes. The polymorphic layer (or hilus) contains a mixed population of glutamatergic mossy cells (MCs) and GABAergic interneurons^5^. Recent experimental work has shown that GCs and MCs form a functional unit to perform pattern separation^6–8^, a neuronal mechanism by which distinct memory traces can be created even if the input pattern is highly overlapping.

The DG is the first node of the classic hippocampal trisynaptic circuit, receiving input from the entorhinal cortex and relaying it to the CA3 via mossy fibers^5,9^. Acting as an intermediary between these two brain regions, the DG plays an important role as a “gate”, filtering input from the entorhinal cortex. This function, often referred to as “Dentate Gating”, is essential for inhibiting and selectively filtering sensory input, preventing excess input from entering other regions of the hippocampus^10^. The impairment of “dentate gating” is implicated in the disruption of normal cortical activity and has often been suggested as a possible cause of seizure activity^11–13^. Thus, the DG plays a pivotal role in the temporal lobe epilepsy^14,15^.

GCs in particular have long been implicated as a major contributor to epileptogenesis due to their strong excitatory effects on pyramidal cells, although some studies have suggested that the abnormal modulation of GCs by MCs may be a cause for pathological conditions^16,17^.

GCaMP6 and newer variants, widely used genetically encoded calcium indicators, have been a reliable sensor capable of detecting even single action potentials^18^. However, it has been recently shown that GCaMP6 expressed in some mouse models may result in aberrant cortical activity^19^. Steinmetz et al. have suggested that GCaMP6 binding to calcium and acting as a calcium buffer affects important cellular functions such as synaptic transmission and gene expression. To investigate the impact of GCaMP6 expression in the various cell types in the DG, we designed a study to test the potential for aberrant activity within the DG, given its critical role in dentate gating and prevention of epilepsy.

In this study, we first performed longitudinal EEG recordings of C57BL/6J mice injected with AAV expressing several variants of GCaMP in the DG and demonstrated that GCaMP6s, but not GCaMP6f or GFP expression induces generalized seizures in mice. We then focused on specific cell types in the dentate gyrus using different Cre lines and showed that GCaMP6s expression in GCs but not MCs or GABAergic neurons reliably evokes seizure activity. Finally, we performed slice electrophysiology and found that GCaMP6s-expressing GCs display increased neuronal excitability, which provides a possible cellular mechanism for seizure generation.

## Results

### Expression of GCaMP6s in DG induces seizures

**Figure 1:**
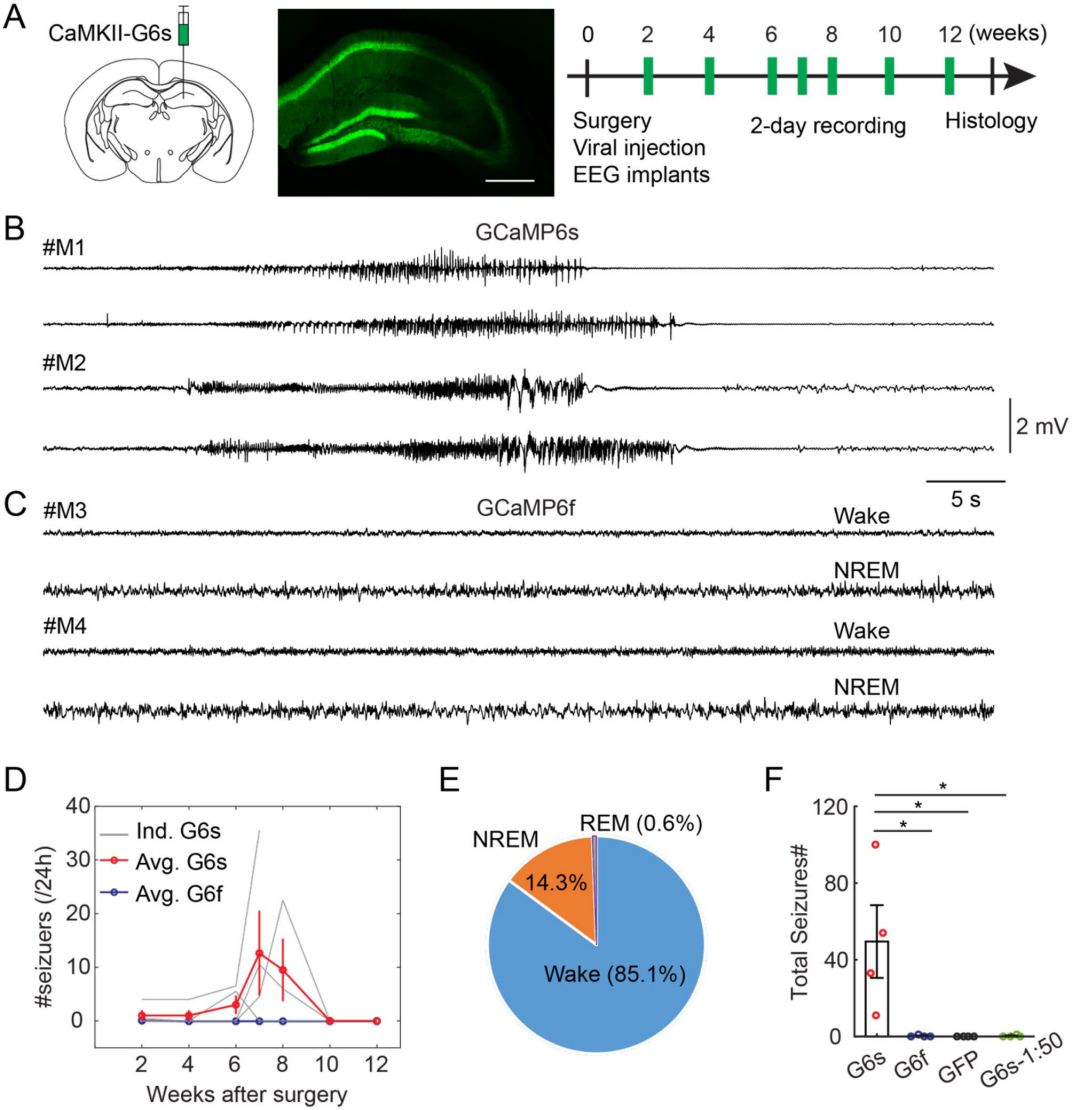
GCaMP6s expression in the hippocampus induces seizures. (**A**) Schematic of experimental design. Left, AAV9-CaMKII-GCaMP6s (G6s) injection in the DG of C57BL/6J mice. Middle, a fluorescent image showing GCaMP6s expression in the hippocampus. Scale bar, 0.5 mm. Right, timeline of EEG recording. (**B**) Representative examples of EEG traces showing seizure events in two mice injected with AAV9-CaMKII-GCaMP6s. (**C**) Representative examples of EEG traces in different brain states in two mice injected with AAV9-CaMKII-GCaMP6f (G6f). (**D**) Quantification of seizure events at different timepoints in mice injected with GCaMP6s (red, N = 4 animals) and GCaMP6f (blue, N = 4 animals). Note that almost no seizures were detected in G6f mice (only 1 event observed at the 2nd week in 1 mouse among 4 recorded mice). (**E**) The percentage of seizure occurrence in different brain states in GCaMP6s mice (averaged data across 4 animals). (**F**) Quantification of total seizure events captured in all recording session in different groups of mice (N = 4 animals for G6s, N = 4 animals for G6f, and N = 4 animals for GFP, *P < 0.05 unpaired t-test).

We unilaterally injected AAV9-CaMKII-GCaMP6s (0.2 µl, Addgene#107790, titer ≥ 1 × 10^13^ vg/mL) in the dorsal DG of C57BL/6J mice (see details in Methods). EEG and EMG electrodes were implanted during surgery to record seizure events and brain states. After two weeks of recovery, we performed longitudinal EEG recordings (Fig. 1A). We observed that GCaMP6s expression in the hippocampus of wild type mice induced generalized tonic–clonic seizures, indicated by EEG signals (Fig. 1B). The seizure events mostly started after the 6th week of viral injection (Fig. 1B,D). Interestingly, the seizure events disappeared in all surviving mice after the 8^th^ week (Fig. 1D, 1 mouse died after the 7th week). The occurrence of seizures is often regulated by wake/sleep states^20,21^. To examine if brain states also affect GCaMP6s-induced seizures, we calculated EEG and EMG signals and detected wake and sleep states in the time window prior to the seizures. Our analysis showed that GCaMP6s-induced seizures predominately occurred during wakefulness (Fig. 1E).

Previous studies suggested that GCaMP6 expression might induce epileptiform activity in the cortex by acting as a calcium buffer^19^. Since different variants of GCaMP6 have different calcium binding properties^18^, we reasoned that different GCaMP6 sensors could have different effect on seizures. To test this, we injected AAV9-CaMKII-GCaMP6f (0.2 µl, Addgene#100,834, titer ≥ 1 × 10^13^ vg/mL), the fast variant of GCaMP6, in the DG and performed similar recordings. Strikingly, we didn’t observe any seizure events in mice expressing GCaMP6f (Fig. 1C,D,F). As another control, we injected AAV9-CaMKII-eGFP (0.2 µl, Addgene#105541, titer ≥ 1 × 10^13^ vg/mL) in the DG. No seizure events were detected over 12 weeks in mice expressing eGFP (Fig. 1F). After recording, we perfused the mice and examined the viral expression in the hippocampus. *Posthoc* histology data demonstrated that CaMKII-driven GCaMP6 was mostly expressed in the GCs of the DG, but notably some in the pyramidal cells of CA1 and CA3 (Fig. 1A). A recent study reported that AAV (AAV1-CAG-FLEX-EGFP) eliminates adult-born dentate granule cells in a dose-dependent manner^22^. Similarly, they observed a loss of immature neuron marker doublecortin (DCX) 2 weeks after the delivery of calcium indicates (AAV8-CaMKII-NES-jRGECO). However, they didn’t characterize the effect on seizures or aberrant activity. To examine whether virus concentration has any effect on seizures, we injected diluted AAV9-CaMKII-GCaMP6s (1:50 dilution) in the DG and repeated the longitudinal EEG experiment. Only 1 seizure event was observed at the 7th week in 1 mouse among 4 mice recorded across 12 weeks (Fig. 1F). This data suggested that a high level of GCaMP6s expression caused by high concentration of AAV is required for seizures.

Together, our results indicate that GCaMP6s, but not GCaMP6f expression in the DG of wildtype mice is sufficient to induce tonic–clonic seizures.

### AAV serotypes and GCaMP variants

**Figure 2:**
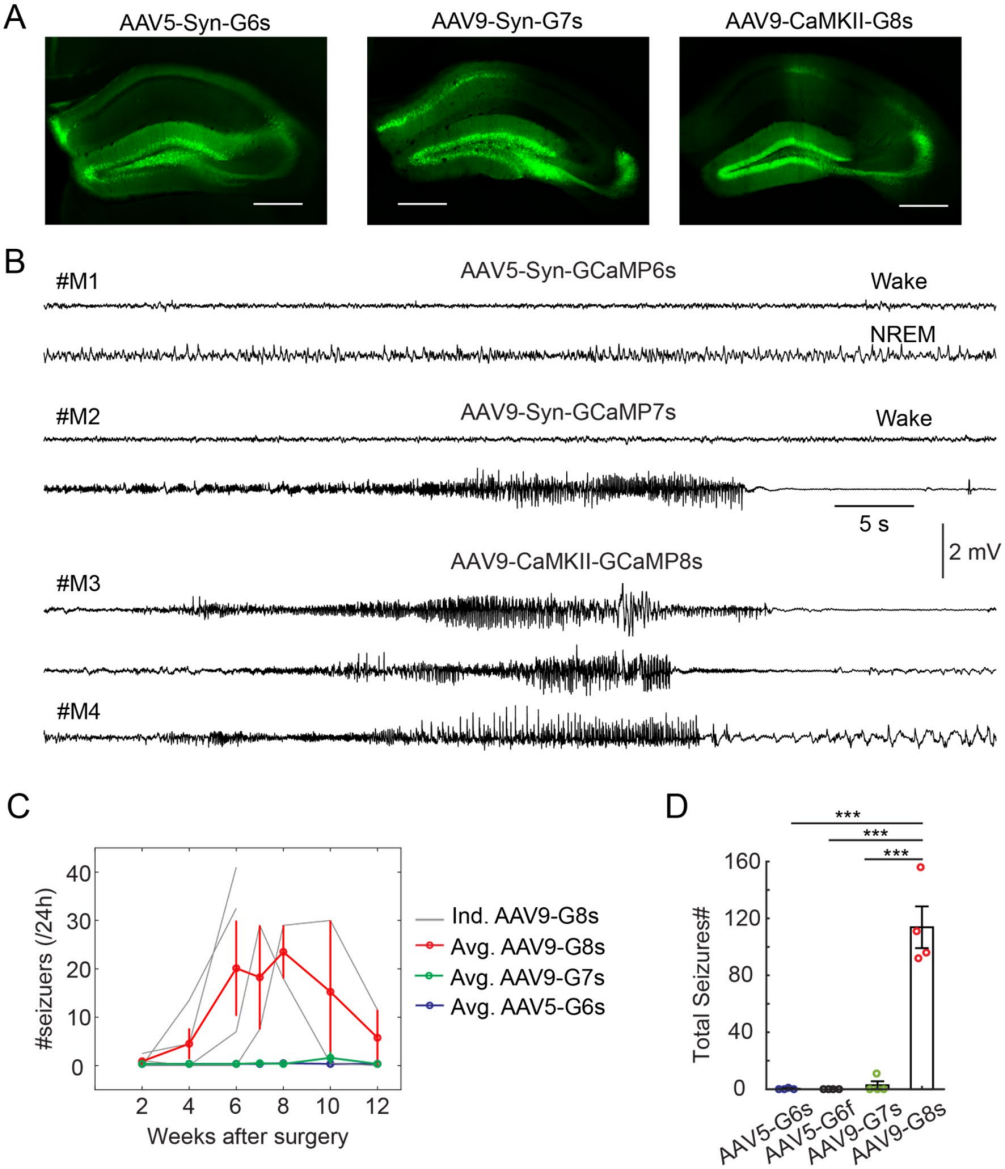
The effect of AAV serotype and GCaMP variant on seizures. (**A**) Fluorescent images showing expression of GCaMP variants in the hippocampus. Scale bar, 0.5 mm. (**B**) Representative examples of EEG traces in mice injected with different viruses. (**C**) Quantification of seizure events at different timepoints in mice injected with AAV9-CamKII-jGCaMP8s (AAV9-G8s, red, N = 4 animals), AAV9-Syn-jGCaMP7s (AAV9-G7s, green, N = 4 animals), and AAV5-Syn-GCaMP6s (blue, N = 4 animals). Note that almost no seizures were detected in AAV5-Syn-GCaMP6s mice (only 1 event observed at the 8^th^ week in 1 mouse among 4 recorded mice). (**D**) Quantification of total seizure events captured in all recording session in different groups of mice (N = 4 animals for AAV5-G6s, N = 4 animals for AAV5-Syn-GCaMP6f, N = 4 animals for AAV9-Syn-jGCaMP7s, and N = 4 animals for AAV9-CaMKII-jGCaMP8s, ***P < 0.001 unpaired t-test).

AAV serotype affects its efficacy to infect neuronal cell types^23^. To test the effect of AAV serotype on seizures, we injected AAV5-Syn-GCaMP6s (0.2 µl, Addgene#100843, titer ≥ 7 × 10^12^ vg/mL) and AAV5-Syn-GCaMP6f (0.2 µl, Addgene#100837, titer ≥ 7 × 10^12^ vg/mL) in the DG (Fig. 2A) and then performed the longitudinal EEG experiments. Interestingly, we observed almost no seizures in both cases (only 1 seizure event at the 8th week in 1 mouse injected with AAV5-Syn-GCaMP6s; 0 seizures in mice injected with AAV5-Syn-GCaMP6f, Fig. 2B–D). These data suggested that the serotype might has a role in seizures. However, a caveat here is the promoter. Due to the unavailability of the CaMKII promoter for AAV5-GCaMP6s, we used the Syn promoter in this study. The promoter likely influences the expression level of GCaMP, which might further affect seizures.

After GCaMP6, newer GCaMP variants have been developed, notably jGCaMP7^24^ and jGCaMP8. To test whether these new variants also induce seizures, we injected AAV9-Syn-jGCaMP7s (0.2 µl, Addgene#104487, titer ≥ 1 × 10^13^ vg/mL) and AAV9-CaMKIIa-jGCaMP8s (0.2 µl, Addgene#176752, titer ≥ 1 × 10^13^ vg/mL) in the DG (Fig. 2A). In one mouse injected with AAV9-Syn-jGCaMP7s (among 4 mice), we observed 11 total seizure events (1 event at the 7^th^ week, 10 events at the 10^th^ week, Fig. 2B–D. No seizures were detected in other 3 mice injected with jGCaMP7s. In contrast, we observed severe seizure events in every mouse injected with AAV9-CaMKIIa-jGCaMP8s (Fig. 2B–D). Two mice died after the 6^th^ week (82 and 65 events over 2 days at the 6^th^ week, respectively). The other two mice survived, but also showed multiple seizure events (Fig. 2C). On average, there were 114 seizure events recorded in mice injected with AAV9-CaMKIIa-jGCaMP8s across the 12-week period (Fig. 2D), which is the double amount of seizures observed in mice injected with AAV9-CaMKII-GCaMP6s (Fig. 1F). Notably, the intermediate variant jGCaMP7s induced very few seizures, which might be due to its Syn promoter. Together, our data suggest that AAV serotype, promoter, and GCaMP variants all contribute to seizure generation in the DG.

### Cell type specific expression

Next, we sought to identify the specific cell types that are involved in seizures. Due to the role of glutaminergic cells in dentate gating, we hypothesize that GCaMP6s expression in excitatory glutaminergic cells in the DG would result in epileptiform cortical activity. To test this, we unilaterally injected AAV1-Syn-Flex-GCaMP6s (0.2 µl, Addgene#100845, titer ≥ 1 × 10^13^ vg/mL) in Cre transgenic mice with known cell-type specific expression patterns. We chose Dock10-Cre^25^, Drd2-Cre^26,27^, and Gad2-Cre^28,29^ to specifically target GCs, MCs, and GABAergic cells in the DG respectively. EEG and EMG electrodes were also implanted during surgery to detect seizure events.

**Figure 3:**
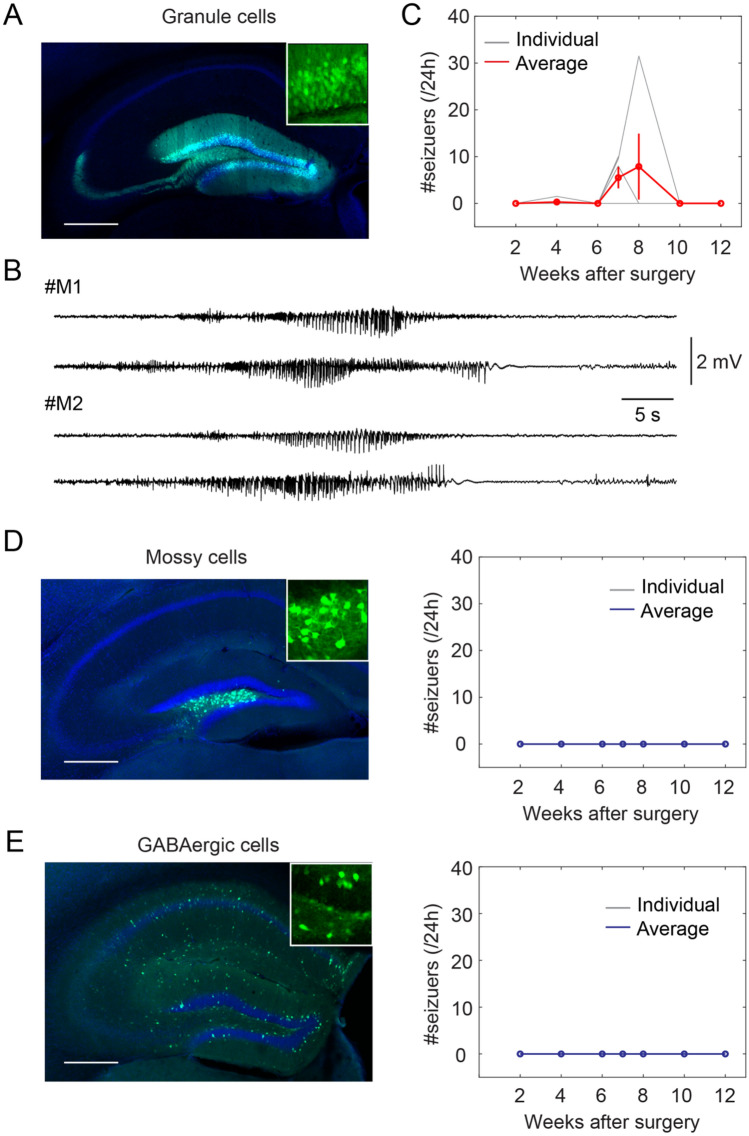
Cell type specific expression of GCaMP6s in the DG. (**A**) Fluorescent image showing GCaMP6s expression in the granule cells of Dock10-Cre mouse injected with AAV1-FLEX-GCaMP6s. Inset, enlarged view of GCaMP6s expression. Scale bar, 0.5 mm. (**B**) Representative examples of EEG traces showing seizure events in two Dock10-Cre mice injected with AAV1-FLEX-GCaMP6s. (**C**) Quantification of seizure events at different timepoints in Dock10-Cre mice (N = 5 animals). (**D**) Left, fluorescent image showing GCaMP6s expression in the mossy cells of Drd2-Cre mouse injected with AAV1-FLEX-GCaMP6s. Inset, enlarged view of GCaMP6s expression. Scale bar, 0.5 mm. Right, Quantification of seizure events at different timepoints in Drd2-Cre mice (N = 5 animals). (**E**) Left, fluorescent image showing GCaMP6s expression in the GABAergic cells of Gad2-Cre mouse injected with AAV1-FLEX-GCaMP6s. Inset, enlarged view of GCaMP6s expression. Scale bar, 0.5 mm. Right, Quantification of seizure events at different timepoints in Gad2-Cre mice (N = 4 animals).

Then, we longitudinally recorded the mice over 12 weeks after surgery. We observed generalized tonic–clonic seizures between the 6–8^th^ week in the Dock10-Cre mice expressing GCaMP6s (Fig. 3A–C, 1 mouse died after the 7^th^ week), similar to that in wildtype mice (Fig. 1). Interestingly, we didn’t observe any seizure events in both Drd2-Cre and Gad2-Cre mice expressing GCaMP6s (Fig. 3D,E). The observation of seizure events in the Dock10-Cre group is in line with our hypothesis that GCaMP6s modulation of electrical activity would stem from the GCs, due to the excitatory nature of the GCs and the GCs making up the bulk of the DG cells. Together, our results demonstrate that GCaMP6s expression in GCs results in generalized seizures.

### Neural correlates of seizures in the dentate gyrus

**Figure 4:**
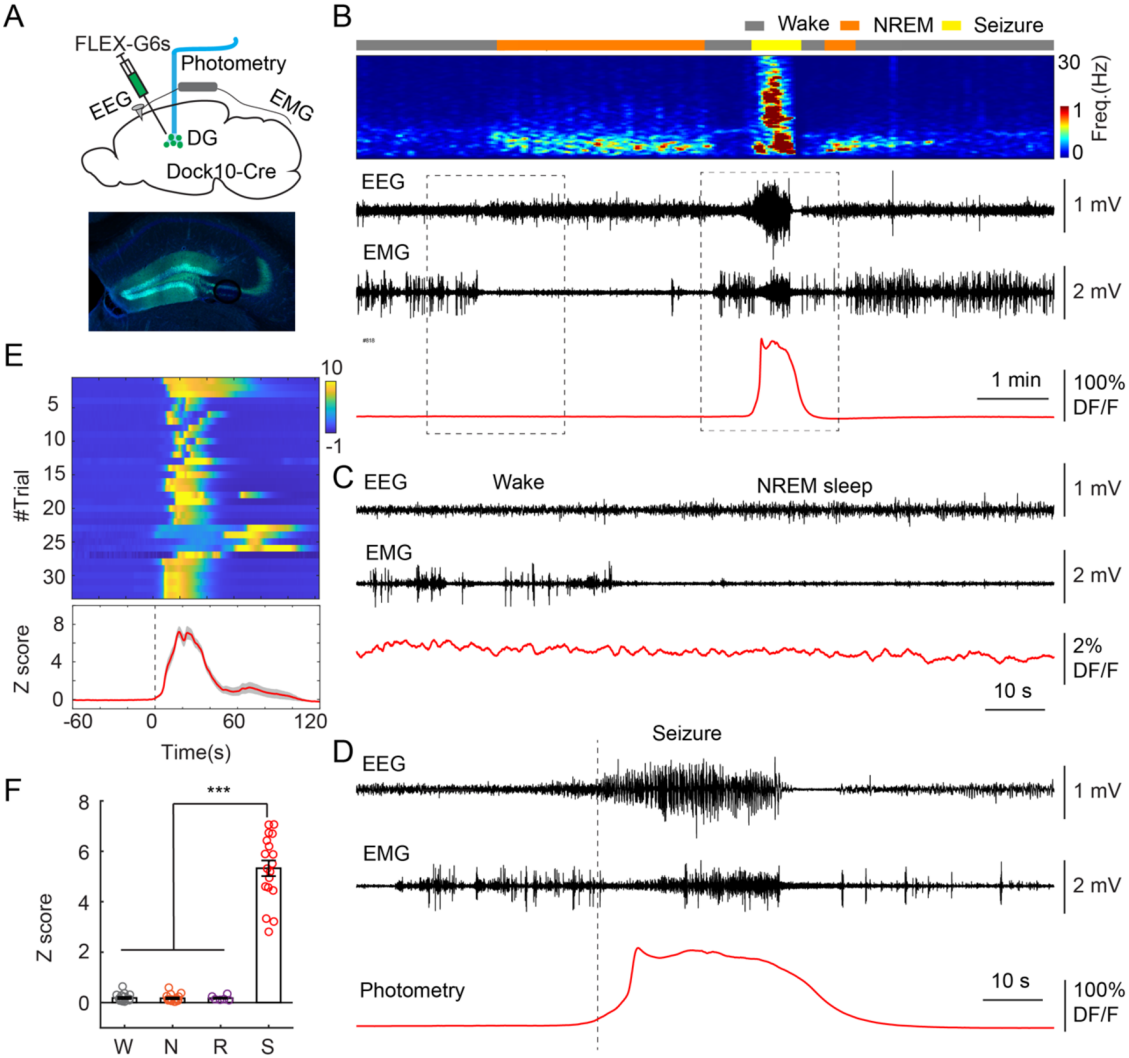
Neural activity of DG in seizures. (**A**) Upper, Schematic of experimental design. Bottom, fluorescent image showing GCaMP6s expression in the GCs of the DG. (**B**) a representative example showing neural activity during a recording session. From top to bottom, color-coded brain states, EEG spectrogram (0–30 Hz), EEG trace, EMG trace, and photometric trace. (**C**) Enlarged view of spontaneous GC activity in wake/sleep baseline. (**D**) Enlarged view of GC activity during a seizure event. (**E**) Top, color-coded GC activity during seizures (34 events from 7 Dock10-Cre animals). Bottom, averaged photometric signals across all events. Time 0 indicates the seizure onset. The activity was normalized to Z scores. (**F**) Quantification of calcium activity in different brain states (W = wake, N = NREM, R = REM, S = seizure, ***P < 0.001, paired t-test, P = 6E−12 between wake and seizure, P = 5E−12 between NREM and seizure, P = 0.00016 between REM and seizure, 18 recording sessions from 7 Dock10-Cre animals). Each dot indicates one 1-h recording session, which contains at least one seizure event.

How does viral expression of GCaMP6s in the DG cause generalized seizures in the cortex? From the perspective of neural circuits, one reasonable hypothesis is that the aberrant activity first generated in the DG cells, then spreads to the cortex. To examine the activity of the GCs in the DG, we combined fiber photometry and EEG recording. We injected AAV1-FLEX-GCaMP6s (0.2 µl, Addgene#100845, titer ≥ 1 × 10^13^ vg/mL) in the DG of Dock10-Cre mice and implanted an optic fiber above the injection site (Fig. 4A). After 2 weeks of recovery, we recorded EEG and photometric signals while animals were experiencing wake/sleep cycles. Multiple sessions were recorded to capture seizure events. Notably, seizure events were typically detected during the 2–4 weeks after surgery in the fiber photometry experiments (data not shown), which is earlier compared to that in EEG experiments described above. This might be due to the additional brain injury caused by the implantation of optic fiber. We should stress that this detrimental effect might be specific to certain brain regions. We performed similar fiber photometry/EEG recordings in various brain regions (e.g. the sensory thalamus, the basal forebrain, the preoptic area, the amygdala) and typically no seizures were observed (data not shown). By aligning photometric signals with EEG signals, we found that GCs generated massive calcium responses during seizures, compared to spontaneous activities during wake/sleep states (Fig. 4B–F). These ictal responses typically had four phases: a slow build-up, a rapid increase to peak, a long plateau, and a decline (Fig. 4D). While the build-up period varied in some cases, this response pattern was largely repeatable in multiple seizures events captured in different mice (Fig. 4E). A notable exemption was particularly long build-up periods (~ 1 min) of seizure-evoked responses in 2 mice (Trial#23–26, among 33 events recorded from 7 mice, Fig. 4E), in which relatively small initial responses were still there, but the calcium peaks arrived at the late stage of seizure events. Together, these GC epileptiform activities correlate with cortical seizure events, suggesting its role in seizure generation.

### Calcium signaling and neuronal excitability

**Figure 5:**
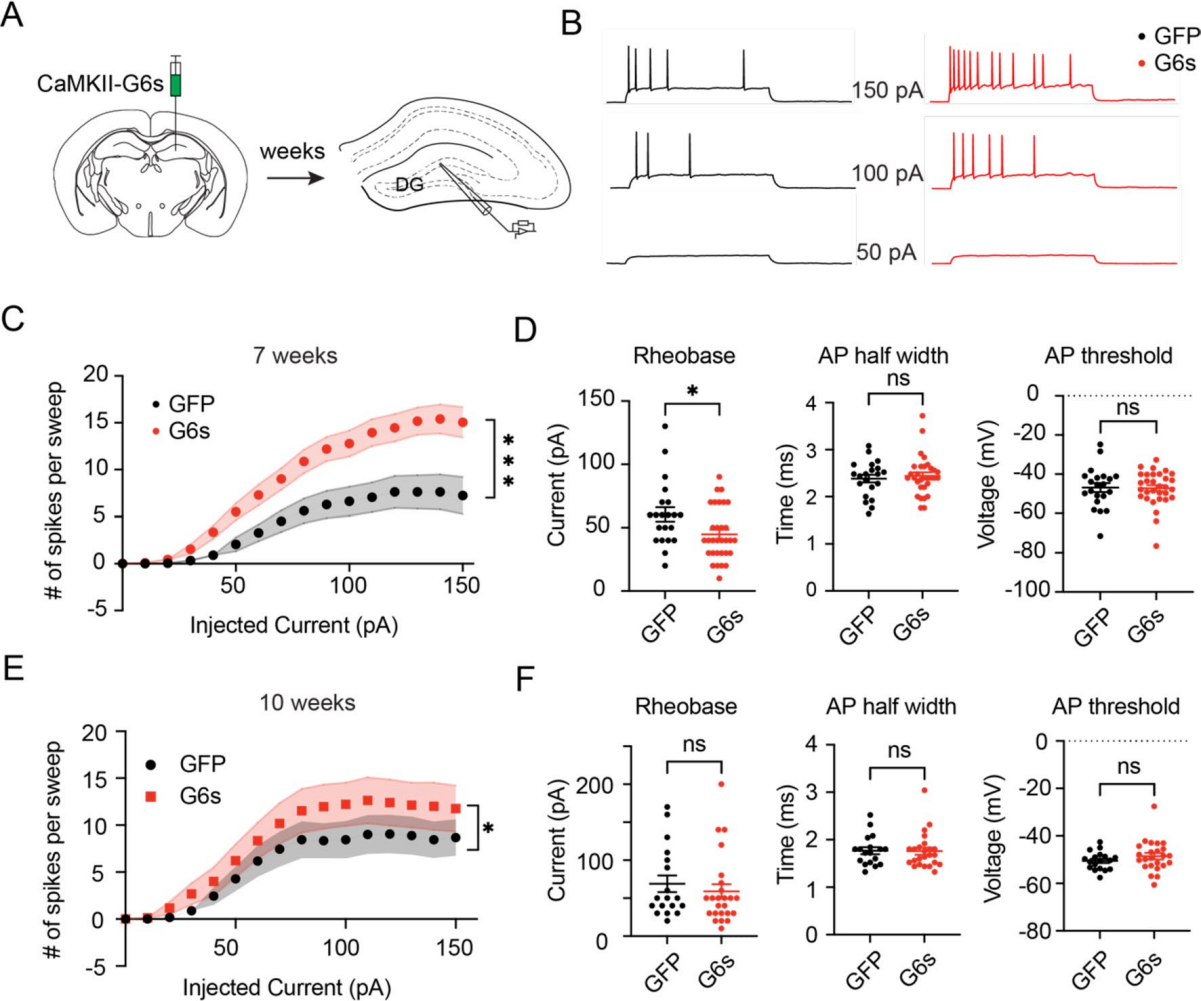
GCaMP6s expression increases neuronal excitability in the DG. (**A**) schematic of experimental design. AAV9-CaMKII-GCaMP6s was injected into the DG of C57BL/6J mice. After 7 weeks, the brain slices containing the dorsal DG were prepared for electrophysiological recording. (**B**) Representative whole-cell current clamp traces of action potentials (APs) fired in response to increasing levels of current stimuli in GFP control (black) and GCaMP6s (G6s, red) dentate gyrus cells. (**C**) F/I curves showing action potentials per stimulation epoch for each current amplitude in GFP control (black, n = 21 cells from N = 4 animals) and GCaMP6s (red, n = 31 cells from N = 3 animals) cells at the 7th week after viral injection. Statistical significance was tested using the slope of the F/I curves which was calculated from a linear regression model (***P < 0.0001, Welch’s t-test). (**D**) Quantification of AP current rheobase, half width, and voltage threshold in two groups (*P < 0.05, ns no significance, Mann–Whitney test). (**E**) F/I curves in GFP control (black, n = 18 cells from N = 3 animals) and GCaMP6s (red, n = 24 cells from N = 3 animals) cells at the 10th week after viral injection. Statistical significance was tested using the slope of the F/I curves which was calculated from a linear regression model (*P = 0.0127, Welch’s t-test). (**F**) Quantification of AP current rheobase, half width, and voltage threshold in two groups (ns no significance, Mann–Whitney test).

Next, we explored the cellular mechanism that could underlie GCaMP6s-induced seizures. Given the ability of GCaMP6s to buffer intracellular calcium, we reasoned that GCaMP6s expression in the GCs affects neuronal excitability through calcium-related pathways^30^. To test this hypothesis, we performed slice electrophysiology in C57BL/6J mice injected with AAV9-CaMKII-GCaMP6s and AAV9-CaMKII-GFP viruses. Based on the timeline of in vivo seizure development (Fig. 1), we recorded brain slices prepared at the 7^th^ and 10^th^ week after the viral injection (Fig. 5A). We predicted that the excitability in the GCaMP6s-expressing cells would increase significantly at the 7^th^ week, then recover at the 10^th^ week, compared to that in the GFP-expressing cells. To test these, we current-clamped GCs in the DG and injected steps to elicit action potentials (APs). Strikingly, we found that current injection consistently evoked more APs in GCaMP6s-expresing GCs at the 7^th^ week timepoint, compared to that expressing GFP (Fig. 5B,C). In addition, GCaMP6s cells showed a slight decrease in rheobase and no significant changes in AP half width and voltage threshold relative to those of GFP mice (Fig. 5D). At the 10^th^ week timepoint, the GCaMP6s cells exhibited slightly higher excitability than the GFP control cells (Fig. 5E), but the difference between GCaMP6s and GFP groups is much smaller, compared to that in the 7^th^ week. We also didn’t observe any significant difference between groups for rheobase, AP threshold, and AP half width (Fig. 5F). Together, our data demonstrated that GCaMP6s expression in the GCs increases neuronal excitability, which might induce hyperactivity in the DG and eventually epileptiform activity in the cortex.

## Discussion

GCaMP6 and newer variants are widely used calcium sensors for functional calcium imaging in the field of neuroscience. It is often considered that the sensor does not affect cellular firing and as such is a good measure of neuronal activity. In this study, we show that the slow versions of GCaMP, specifically GCaMP6s and jGCaMP8s, can induce epileptiform electrical activity in mice when expressed in a population of DG neurons. Our findings are in accordance with a previous study by Steinmetz et al. which showed that GCaMP6 evoked aberrant cortical activity in some transgenic mice lines^19^. Certainly, the big difference is that more brain regions are likely affected in GCaMP6 transgenic mice, while our viral approach selectively targets one brain region. Nevertheless, their study alongside ours shows that GCaMP6s may have a profound impact on modulating neuronal activity. Importantly, our study revealed the timeline of epileptiform activity after viral injection of GCaMP6s and other variants in different AAV serotypes, which provides valuable information to researchers in calcium imaging to better design their experimental schedule.

The mechanism behind the ability of GCaMP6s or jGCaMP8s to modulate neuronal activity is still unknown. Our GFP control experiments suggest that inflammation and tissue damage caused by the viral delivery method is not a contributing factor. We speculate that GCaMP6s and jGCaMP8s ability to modulate neuronal activity is due to its excessive binding of Ca^2+^ and acting as a Ca^2+^ buffer intracellularly^31^. Calcium has numerous significant functions in neurons, such as regulating calcium-dependent receptor or ion channel function, synaptic transmission, expression of genes related to synaptic plasticity^32,33^. Another possibility is that GCaMP can directly affects calcium channels. Indeed, a recent study shows that GCaMP interferes with both gating and signaling of L-type calcium channels (Ca_V_1) via the calmodulin unit^34^. Nevertheless, any disturbance in calcium-related functions can potentially cause changes in network activity. As evidence, certain genetic mutations to calcium channels in mice have been linked to epilepsy^35^. Furthermore, the loss of the calcium binding protein from dentate gyrus cells has been observed in both animal models of epilepsy^36,37^ and human temporal lobe epilepsy^38^. Interestingly, although having a similar mechanism to GCaMP6s and also binding to Ca^2+^, our study showed that GCaMP6f expression in the DG was not sufficient to induce seizure events in C57 mice, which suggest that Ca^2+^ binding properties of the sensor has a big impact on epileptogenesis. One possibility is that GCaMP6f does not elicit a strong enough buffer effect to modulate cellular activity, due to its fast calcium binding dynamics^18^. Notably, Steinmetz et al. showed that GCaMP6f expression in some transgenic lines was able to induce aberrant cortical activity, even generalized tonic–clonic seizures^19^. These findings suggest that different cell types might respond differently to the disturbance of intracellular calcium buffering.

Using different transgenic Cre lines, we showed that specific cell type expression of GCaMP6s in granule cells of the DG is sufficient to produce seizure events. In wildtype mice, AAV likely spreads to other hippocampal areas, such as CA1 (Fig. 1A). We cannot exclude the possibility of the involvement of CA1 neurons in seizures. However, the dense organization and excitatory nature of the GCs are well-suited to drive the network activity beyond the physiological state when aberrant activity is introduced. Previous studies reported the involvement of the MCs in seizures^16,17^. For instance, Botterill et al. showed that chemogenetic inhibition of MCs during severe seizures reduced manifestations those seizures while optogenetic activation of MCs was pro-convulsant^17^. In our case, GCaMP6s expression in MCs didn’t induce spontaneous seizures. This might be due to the different intrinsic calcium signaling and the ability to excite the hippocampal network between the GCs and MCs. Moreover, our data doesn’t exclude the possibility of the recruitment of MCs in GCs-induced seizures. In another study, Spampanato and Dudek reported that targeted interneuron ablation using the diphtheria toxin receptor (DTR) in the hippocampus of Gad2-Cre mice causes spontaneous seizures^29^. In our study, GCaMP6s expression in GABAergic neurons didn’t induce any seizures. We speculate that different mechanisms might be involved, as ablation of GABAergic neurons could result in more dramatic changes of network activity within the hippocampus.

Another interesting result we observed was that seizure events in mice seemed to be aggregating at the period of 6–8 weeks after viral injection. The reason for this aggregation is still unknown. We speculate that such an effect may have resulted from the accumulation of the GCaMP6s protein in the DG over a period of time. We should point out that seizure events are detected at the cortical level, reflecting a global change of brain activity. GCaMP6s-induced aberrant activity could occur much earlier in the DG. Then, the epileptiform activity could spread slowly from the DG to the cortex via the hippocampo-cortical circuits. Surprisingly, seizure events mostly disappeared after the 8^th^ week in both C57BL/6J and Dock10-Cre mice expressing GCaMP6s. We speculate that the toxic effect might be mitigated by certain cellular mechanisms of protein homeostasis. More work is required to investigate this phenomenon.

## Methods

### Animals

All procedures were carried out in accordance with the US National Institute of Health (NIH) guidelines for the care and use of laboratory animals, and approved by the Animal Care and Use Committees of Columbia University. The following mouse lines were used in the current study: C57BL/6J (JAX 000664), Dock10-Cre, Drd2-Cre, Gad2-IRES-Cre (JAX 028867). For C57BL/6J, only male adult mice (8–10 weeks at the time of surgery) were used. For Cre lines, both male and female adult mice (8–10 weeks at the time of surgery) were used for all experiments.

The design of the study and the methods used are reported in accordance with ARRIVE guidelines (https://arriveguidelines.org/arrive-guidelines).

### Viral constructs

All viruses were obtained from Addgene. Detailed information is listed below:

**Table:** 

AAV-name	Addgene-cat#	Titer	Variant	Promotor	Serotype	PI/lab
AAV.CamKII.GCaMP6s.WPRE.SV40	107790-AAV9	≥ 1 × 10^13^ vg/mL	GCaMP6s	CamKII	AAV9	James M. Wilson
AAV.CamKII.GCaMP6f.WPRE.SV40	100834-AAV9	≥ 1 × 10^13^ vg/mL	GCaMP6f	CamKII	AAV9	James M. Wilson
AAV.CamKII0.4.eGFP.WPRE.rBG	105541-AAV9	≥ 1 × 10^13^ vg/mL	eGFP	CamkII	AAV9	James M. Wilson
pAAV.Syn.Flex.GCaMP6s.WPRE.SV40	100845-AAV1	≥ 1 × 10^13^ vg/mL	GCaMP6s	Synapsin	AAV1	Douglas Kim
pAAV.Syn.GCaMP6f.WPRE.SV40	100837-AAV5	≥ 7 × 10^12^ vg/mL	GCaMP6f	Synapsin	AAV5	Douglas Kim
pAAV.Syn.GCaMP6s.WPRE.SV40	100843-AAV5	≥ 7 × 10^12^ vg/mL	GCaMP6s	Synapsin	AAV5	Douglas Kim
pGP-AAV-syn-jGCaMP7s-WPRE	104487-AAV9	≥ 1 × 10^13^ vg/mL	jGCaMP7s	Synapsin	AAV9	Douglas Kim
AAV-CamKIIa-jGCaMP8s-WPRE	176752-AAV9	≥ 1 × 10^13^ vg/mL	jGCaMP8s	CamKIIa	AAV9	Loren Looger

### Surgical procedures

Mice were anaesthetized with a mixture of ketamine and Xylazine (100 mg kg^−1^ and 10 mg kg^−1^, intraperitoneally), then placed on a stereotaxic frame with a closed-loop heating system to maintain body temperature. After asepsis, the skin was incised to expose the skull and a small craniotomy (~ 0.5 mm in diameter) was made on the skull above the regions of interest. A solution containing ~ 200 nl viral construct was loaded into a pulled glass capillary and injected into the dorsal dentate gyrus (AP − 1.9 mm, ML 1.4 mm, DV 1.8 mm) using a Nanoinjector (WPI). The DV Coordinates are relative to the pial surface. For EEG and EMG recordings, a reference screw was inserted into the skull on top of the cerebellum. EEG recordings were made from two screws on top of the cortex: (1) 1 mm from midline and 1.5 mm anterior to the bregma, (2) 1 mm from midline and 1.5 mm posterior to the bregma. Two EMG electrodes were bilaterally inserted into the neck musculature. EEG screws and EMG electrodes were connected to a PCB board which was soldered with a 5-position pin connector. All the implants were secured onto the skull with dental cement (Lang Dental Manufacturing). After surgery, the animals were returned to home-cage to recover for at least two weeks before any experiment.

### EEG recording and analysis

Mouse seizure and sleep behavior were monitored using EEG and EMG recording along with an infrared video camera at 30 frames per second. Recordings were performed for 24–48 h (light on at 7:00 am and off at 7:00 pm) in a behavioral chamber inside a sound attenuating cubicle (Med Associated Inc.). Animals were habituated in the chamber for at least 4 h before recording. EEG and EMG signals were recorded, bandpass filtered at 0.5–500 Hz, and digitized at 1017 Hz with 32-channel amplifiers (TDT, PZ5 and RZ5D or Neuralynx Digital Lynx 4S). For sleep analysis, spectral analysis was carried out using fast Fourier transform (FFT) over a 5 s sliding window, sequentially shifted by 2 s increments (bins). Brain states were semi-automatically classified into wake, NREM sleep, and REM sleep states using a custom-written MATLAB program (wake: desynchronized EEG and high EMG activity; NREM: synchronized EEG with high-amplitude, delta frequency (0.5–4 Hz) activity and low EMG activity; REM: high power at theta frequencies (6–9 Hz) and low EMG activity). Semi-automated classification was validated manually by trained experimenters.

For seizure analysis, FFT of EEG was performed as described above. Then, the “seizure”-power (19–23 Hz) was calculated to extract seizure events based on a threshold of 2–3 standard deviations. We chose the 19–23 Hz band to detect seizures based on its clear separation from normal brain oscillatory activities. Algorithm-detected seizure events were further reviewed by trained experimenters.

### Fiber photometry

Fiber photometry recordings were performed essentially as previously described^39^. In brief, Ca^2+^ dependent GCaMP fluorescence was excited by sinusoidal modulated LED light (465 nm, 220 Hz; 405 nm, 350 Hz, Doric lenses) and detected by a femtowatt silicon photoreceiver (New Port, 2151). Photometric signals and EEG/EMG signals were simultaneously acquired by a real-time processor (RZ5D, TDT, sampling rate of 1017 Hz) and synchronized with behavioral video recording. A motorized commutator (ACO32, TDT) was used to route electrical wires and optical fiber. The collected data were analyzed by custom MATLAB scripts. They were first extracted and subjected to a low-pass filter at 20 Hz. A least-squares linear fit was then applied to produce a fitted 405 nm signal. The DF/F was calculated as: (F-F0)/F0, where F0 was the fitted 405 nm signals. Data were smoothed using a moving average method over 0.1 s. To compare activity across animals, photometric data were further normalized using Z-score calculation in each mouse.

### Slice electrophysiology

Mice were anesthetized, and 300um-thick coronal slices containing dentate gyrus were prepared. Brains were sliced in ice-cold sucrose cutting solution (310 mOsm) containing (in mM): sucrose (234), glucose (11), NaHCO_3_ (26), KCl (2.5), NaH_2_PO_4_ (1.25), MgSO_4_ (10), and CaCl_2_ (0.5). Slices were then transferred to artificial cerebrospinal fluid (aCSF) solution (298 mOsm) containing (in mM): glucose (10), NaHCO_3_ (26), KCl (2.5), NaHPO_4_ (1.25), MgSO_4_ (1), CaCl_2_ (2), and NaCl (126). Slices were incubated in aCSF warmed to 35 °C for 40 min and then at room temperature until recording. Slicing solution and aCSF were continuously bubbled with 5%CO^2^/95%O^2^.

Electrophysiological data were acquired using Multiclamp 700B amplifiers (Molecular Devices) via Clampex 10.7 software. Data was acquired at 100 kHz and filtered at 10 kHz. Patch pipettes were pulled with a P-97 pipette puller (Sutter Instruments) using 1.5-mm outer diameter, 1.28-mm inner diameter filamented capillary glass (World Precision Instruments). The resistance of pipettes was 3–5 mΩ.

For current-clamp recordings, the internal solution contained (in mM): K^+^ methanesulfonate (130), Na^+^ methanesulfonate (10), CaCl_2_ (1), HEPES (10), EGTA (10), Mg-ATP (5) and Na2-GTP (0.5). KOH was used to adjust the pH to 7.2. With giga-ohm seal formed and bridge balanced under whole-cell configuration, pipette capacitance was then compensated by 50% of the fast capacitance. Intrinsic electrophysiological properties of neurons were tested by injecting 1000-ms square current pulses incrementing in 10pA-steps. Action potential features were analyzed using Python.

### Histology

Viral expression and placement of optical implants were verified at the termination of the experiments using DAPI counterstaining of 100 μm coronal sections (Prolong Gold Antifade Mountant with DAPI, Invitrogen). Images were acquired using a Zeiss 810 confocal microscope, and processed in ImageJ.

### Statistics

No statistical methods were used to predetermine sample size, and investigators were not blinded to group allocation. No method of randomization was used to determine how animals were allocated to experimental groups. Mice in which post hoc histological examination showed viral targeting or fiber implantation was in the wrong location were excluded from analysis. Paired and unpaired T-test were used and indicated in the respective figure legends. All analyses were performed in MATLAB. Data are presented as mean ± s.e.m.

## Data Availability

All data supporting the findings of this study are available from the corresponding author upon reasonable request.

## Code availability

Custom scripts for EEG/EMG and photometry analysis are available from the corresponding author upon reasonable request.
